# Hexose/pentose ratio in rhizosphere exudates-mediated soil eutrophic/oligotrophic bacteria regulates the growth pattern of host plant in young apple–aromatic plant intercropping systems

**DOI:** 10.3389/fmicb.2024.1364355

**Published:** 2024-03-25

**Authors:** Mengnan Zhao, Yue Sun, Meilin Dong, Kui Zhang, Jie Zhang, Xiaoxiao Qin, Yuncong Yao

**Affiliations:** ^1^College of Biological Sciences and Technology, Beijing Forestry University, Beijing, China; ^2^Beijing Advanced Innovation Center for Tree Breeding by Molecular Design, Beijing University of Agriculture, Beijing, China; ^3^Plant Science and Technology College, Beijing University of Agriculture, Beijing, China; ^4^Beijing Key Laboratory for Agricultural Application and New Technique, Beijing University of Agriculture, Beijing, China

**Keywords:** apple intercropping, interspecific competition, hexose/pentose, eutrophic/oligotrophic bacteria, carbon and nitrogen cycling

## Abstract

**Introduction:**

The positive effect of intercropping on host plant growth through plant–soil feedback has been established. However, the mechanisms through which intercropping induces interspecific competition remain unclear.

**Methods:**

In this study, we selected young apple trees for intercropping with two companion plants: medium growth-potential *Mentha haplocalyx* Briq. (TM) and high growth-potential *Ageratum conyzoides* L. (TA) and conducted mixed intercropping treatment with both types (TMA) and a control treatment of monocropping apples (CT).

**Results:**

Our findings revealed that TM increased the under-ground biomass of apple trees and TA and TMA decreased the above-ground biomass of apple trees, with the lowest above-ground biomass of apple trees in TA. The above- and under-ground biomass of intercrops in TA and TMA were higher than those in TM, with the highest in TA, suggesting that the interspecific competition was the most pronounced in TA. TA had a detrimental effect on the photosynthesis ability and antioxidant capacity of apple leaves, resulting in a decrease in above-ground apple biomass. Furthermore, TA led to a reduction in organic acids, alcohols, carbohydrates, and hydrocarbons in the apple rhizosphere soil (FRS) compared to those in both soil bulk (BS) and aromatic plant rhizosphere soil (ARS). Notably, TA caused an increase in pentose content and a decrease in the hexose/pentose (C6/C5) ratio in FRS, while ARS exhibited higher hexose content and a higher C6/C5 ratio. The changes in exudates induced by TA favored an increase in taxon members of Actinobacteria while reducing Proteobacteria in FRS compared to that in ARS. This led to a higher eutrophic/oligotrophic bacteria ratio relative to TM.

**Discussion:**

This novel perspective sheds light on how interspecific competition, mediated by root exudates and microbial community feedback, influences plant growth and development.

## Introduction

Intercropping is an effective strategy to enhance grain production, overcome the limitations associated with continuous monocropping, and deliver various ecological benefits (Bai et al., 2022; Li et al., 2023). Over the long term, intercropping not only improves the soil’s nutrient cycle but also confers resistance to diseases, pests, and competitive plant species (Chadfield et al., 2022). This efficiency in complementing the temporal and spatial diversification (Brooker et al., 2015; Jian et al., 2020; Li et al., 2020) of plants has been well-documented in the literature. However, not all intercropping systems yield positive interactive effects on both intercrops and host plants (Corre-Hellou et al., 2011). For instance, intercropping legumes with cereal species has been observed to reduce the biomass of the host plant and result in decreased nitrate fixation compared to single crops (Corre-Hellou et al., 2011). These outcomes arise from the intricate interplay of intraspecific and interspecific interactions among crops chosen for intercropping, driven by differences in the chemical composition of their root exudates and associated microbial activities (Xu et al., 2021; Zhang et al., 2021). These factors can exert significant influence on carbon inputs, sequestration, and nutrient regulation, leading to alterations in the activity, abundance, and diversity of soil microbial communities (Venter et al., 2016). Nevertheless, the specific mechanisms by which these interactions shape chemical changes in root exudates and subsequently impact the performance of soil microbial communities within distinct root zones remain a subject of ongoing research and investigation. The remarkable ability of plant roots to release a wide array of compounds into the rhizosphere is a pivotal feature (Bais et al., 2006). These plant root exudates wield significant influence on various aspects of plant performance, including modifying soil physicochemical properties and nutrient availability, fostering interactions between roots and beneficial microbes, and suppressing harmful pathogens (Berendsen et al., 2012). A prime example of this phenomenon is found in maize, where the compound 2,4-dihydroxy-7-methoxy-2H-1,4-benzoxazin-3(4H)-one (DIMBOA) acts as an allelochemical against soil microbes and neighboring plants, while also serving as a chemoattractant for plant growth-promoting bacterium (Neal et al., 2012). In the context of intercropping systems, the diverse composition of plant litter and root exudates provides an array of decomposition substrates for microbes. This leads to significant shifts in the soil microbial community, thereby enhancing soil nutrient cycling compared to that in monocropping systems (Kim et al., 2020). This underscores the positive interactions occurring between intercrops, host plants, and the associated root microbiota, all of which contribute positively to the soil’s nutrient cycle and the plants thriving within it. However, it is important to note that plants occasionally employ secondary root exudates to manipulate microbial communities to the detriment of neighboring plants. A case in point is the root-secreted phytotoxin catechin, which plays a role in the invasive behavior of knapweed in the rhizosphere. (−)-Catechin exhibits allelochemical activity, whereas (+)-catechin inhibits soil-borne bacteria (Bais et al., 2006), illustrating the complex and sometimes competitive nature of these interactions.

Furthermore, root exudates play a crucial role in regulating plant–plant interactions by indirectly impacting soil composition (Bais et al., 2006). They exert their influence on soil nutrient availability through alterations in soil properties, chemistry, and biological processes. These changes can significantly influence the outcome of resource competition among plants, especially when the soil nutrient are limited (Bais et al., 2006).

In agroforestry systems utilizing intercropping patterns, plant interactions are inevitable (Steinbeiss et al., 2008; Fornara and Tilman, 2009). Therefore, the selection of plant species with synergistic effects and the avoidance of those with negative interactive effects are pivotal for promoting soil health and enhancing cash crop yields (Zhou et al., 2023). However, the differences in root exudate composition and concentration among plant species and developmental stages and under different environmental conditions underscore the importance of careful plant species selection and collocation within intercropping systems (Lian et al., 2020; Jiang et al., 2022).

Plant–microbe interactions can positively impact plant growth through various mechanisms, including the fixation of atmospheric nitrogen (Moulin et al., 2001). The chemical attraction between soil microbes and plant roots initiates cross-talk, which largely dictates specific host-microbe relationships (Bais et al., 2006). For instance, plant flavonoids mediate rhizobia-plant associations in the N-fixing process of legumes, enabling rhizobia to distinguish their host from other legumes (Bais et al., 2006). Intercropping with multiple species, which is the combination of at least two plant species, can offer additional benefits by increasing not only microbial diversity but also the abundance of beneficial soil microbes compared to monocultures (Bever et al., 2015; Xue et al., 2023). This positive effect aligns with the well-established relationship between plant biodiversity, root exudate availability and diversity, and soil microbial diversity (Maron et al., 2011). Moreover, intercropping alters the structure and function of soil microbial communities, fostering increased inter-taxon associations, which in turn leads to a greater number of metabolic pathways associated with nutrient cycling and an abundance of beneficial microbes (Zheng et al., 2018). These advantageous effects are often attributed to the composition of intercrop root exudates as they influence soil microbial communities, facilitate the assembly of beneficial microbes in different root zones, and promote regional nutrient cycling and supply, ultimately contributing to the plant–soil feedback effect (Zhang et al., 2021). For example, intercropping with licorice may improve apple tree growth and disease resistance (Li et al., 2022). However, there is limited information available regarding the negative effects of interactions between intercrops on soil microbial communities and nutrient cycling, particularly under conditions of resource limitation, such as drought, low light, and barren conditions (Bais et al., 2006). Plants select rhizosphere microbial communities through root exudates and the preference of soil microbes for specific root exudates can drive the establishment of rhizosphere microbial communities (Zhalnina et al., 2018). Changes in microbial population structure are a result of root exudates and environmental selection pressures, stemming from intraspecific exchanges and interspecific migrations of microbial populations (Yuan et al., 2018). The competition modes adopted and the mechanisms by which soil microbes are mobilized to participate in the competition between intercrops and host plants at different root zones in agroforestry ecosystems remain poorly understood.

Aromatic plants are a source of cosmetics, essential oils, and biocides (Lubbe and Verpoorte, 2011; Tang et al., 2013; Song et al., 2014). Previous studies have shown that intercropping aromatic plants in orchards improved soil nutrient status, inhibited harmful pests and pathogenic fungi, and enhanced soil microbial community diversity and stability via produced essential oil, aroma chemicals, and alkaloids (Song et al., 2010; Misra et al., 2019; Zhang et al., 2021). In addition, intercropping various plant species, including single and multiple species alongside aromatic plants in apple orchards, resulted in significant variations in soil microbial community diversity and their respective functions, which affected the growth and development of plants (Zhang et al., 2021). For example, basil and summer savory (Labiatae) increased the diversity of the microbial community while ageratum (Compositae) decreased those (Song et al., 2010; Zhang et al., 2021). These suggest that there will be different mechanisms that intercropping with single species and with mixed species to regulate soil microbial community in agroecosystem. Therefore, we chose mint and Ageratum as intercrops in this study. The objective of this study was to design pot experiments, where young apple trees were intercropped with two different aromatic plant species: medium growth-potential *Mentha haplocalyx* Briq. (Labiatae) and high growth-potential *Ageratum conyzoides* L. (Compositae), as well as a mixed intercropping treatment. Additionally, the control group only planted apple trees in pots. This study aims to unveil the interaction mechanisms between root exudates and the dominant soil microbes in the rhizosphere of host-intercrop plants and determine how these interactions provide feedback on the growth patterns of the host plant by investigating (1) how aromatic plants with differing growth potentials compete with the host plant for soil moisture and nutrients, and whether they inhibit or promote the growth of the host plant; (2) how the root exudates of intercrops influence the root exudation of the host plants; and (3) how microbial community in intercrop’s rhizosphere impact the composition and function of the microbial community in the host plant’s rhizosphere.

## Materials and methods

### Materials and plant culture

*Malus hupehensis* Rehd., commonly known as apple rootstock, served as the host plant. Two aromatic plant species were selected as intercrops: mint (*Mentha haplocalyx* Briq.), belonging to Labiatae, an aromatic plant native to Europe and the Mediterranean with wide global distribution, and ageratum (*Ageratum conyzoides* L.), belonging to Compositae, an aromatic plant native to Central and South America and found in abundance in Africa and Southeast Asia, which have different effects on soil microbial communities in previous studies in orchard (Song et al., 2010; Zhang et al., 2021). Additionally, mixed combinations of these aromatic plants were employed as intercrops. The experimental potting has an upper diameter of 21 cm, a lower diameter of 19 cm, a height of 26 cm, and the weight of soil was 8 kg/pot. The bottom of the pot was perforated and covered with three layers of permeable nylon film to prevent soil from flowing out with water. The experimental potting medium was composed of a mixture of peat, perlite, vermiculite, and soil in equal proportions (1,1:1:1, v/v). The soil was collected from an apple orchard located in the Changping District of Beijing, China (115°50′E, 40°23′N) and sieved with 5 mm pore mesh. The soil had a pH of 7.20, soil organic matter (SOM) of 3.98%, total nitrogen (TN) of 1.24 g/kg, total phosphorus (TP) of 0.41 g/kg, total potassium (TK) of 16.83 g/kg, available nitrogen (AN) of 167.39 mg/kg, available phosphorus (AP) of 25.83 mg/kg, and available potassium (AK) of 12.03 mg/kg. For the pot cultivation, 1-year-old seedlings of *M. hupehensis*, approximately 10 cm in height, were selected. Seeds of *M. haplocalyx* and *A. conyzoides* were grown on plastic trays (height: 150 mm, width: 250 mm, length: 250 mm) after being disinfected and soaked (Lian et al., 2020). When 70% of the seeds had successfully germinated, the aromatic plant seedlings were transplanted into pre-filled pots, each containing three *M. hupehensis* seedlings with similar growth patterns (Zhang et al., 2021). The pot experiment and germination were conducted under controlled conditions at Beijing University of Agriculture (40°09′N, 116°31′E). The cultivation environment: temperature was 23–25°C, light/dark was 16/8 h, and relative humidity was 85%.

### Experimental design

The pot culture experiment was divided into four treatments: monoculture of three young annual *M. hupehensis* trees in a 10 cm equilateral triangle pattern (CT); intercropping of three young annual *M. hupehensis* trees with 10 *M. haplocalyx* seedlings (TM); intercropping of three young annual *M. hupehensis* trees with 10 *A. conyzoides* seedlings (TA); and mixed intercropping of three young annual *M. hupehensis* trees with five *M. haplocalyx* seedlings and five *A. conyzoides* seedlings (TMA). Randomized block experimental design was used with three replicates, and each experimental plot contained 10 pots (Supplementary Figure S1).

### Soil and plant sampling

At 120 days following the intercropping treatment, the rhizosphere and bulk soils of apple trees and intercrops were sampled following the protocol outlined by Niu et al. (2017). Briefly, we carefully took out host plants and intercrops, shook off the loosely adhering bulk soil, and collected the tightly adhering rhizosphere soil using a brush. The bulk soil and rhizosphere soil were passed through a 4 mm sieve. The collected soil samples were preserved at −80°C to facilitate total DNA extraction and GC–MSD analysis.

Plant samples were collected on the 120-day after the intercropping treatment and divided into their above-ground and below-ground components for subsequent physiological measurements.

### Physiological and biochemical analysis of plant traits

The determination of plant biomass involved measuring the dry weight of the plants after they had been dried at 65°C. The N levels were determined through the Kjeldahl method (Hirota et al., 2005). Phosphorus (P) and potassium (K) levels were determined using HNO_3_-HClO_4_ (5:1 v/v) and the mass fraction of each element was subsequently quantified using inductively coupled plasma atomic emission spectrometry (Agilent Technologies, Palo Alto, CA, United States; Sanchez et al., 2017). Chlorophyll a and b contents were determined utilizing an acetone–ethanol mixture leaching method (Sinnecker et al., 2002). To quantify soluble proteins, the Coomassie brilliant blue method was employed (Kielkopf et al., 2020). Malondialdehyde (MDA), superoxide dismutase (SOD), catalase (CAT), and peroxidase (POD) levels were analyzed using assay kits [Beijing Solaribio Life Sciences Company (Beijing, China)] following the manufacturer’s instructions.

### Collection of root exudates and GC–MSD analysis

We used GC-MSD to determine the effects of the main components in the root exudate of aromatic plants, including essential oils, aromatic compounds, and alkaloids, on the apple trees (Misra et al., 2019). The stored rhizosphere soil and bulk soil samples (200 mg) were thawed on ice and root exudates were extracted using 1 mL of 50% methanol buffer (methanol:deionized water = 1:1) (Zhang et al., 2021). Briefly, the mixed solution was vortexed for 1 min, subjected to ultrasound for 20 min (on ice), and centrifuged (4°C, 24,000 *g*, 15 min). Thereafter, 200 μL of extracted supernatant mixture samples were analyzed using an Agilent 5975C MSD mass spectrometer coupled to an Agilent 7890A GC system (Agilent Technologies, Palo Alto, CA, United States) according to the manufacturer’s instructions. Briefly, the liquid injection was done using a PAL System RSI 85 (PAL, Lake Elmo, MN, United States). The injector temperature was 230°C; the MS transfer line was 300°C. Separation was performed using an HP-5MS 30 m, 0.25 mm, and 0.25 μm capillary column (Agilent Technologies, Palo Alto, CA, United States) at constant flow 1.5 mL × min^−1^ of helium as a carrier gas. One microliter of the derivatized sample was injected into the injector operating in splitless mode. The temperature of the column was initially set to 80°C and increased to 175°C at a rate of 15°C × min^−1^, followed by an increase to 220°C at 5°C × min^−1^, and a final increase to 320°C at 25°C × min^−1^ (Lopez-Guerrero et al., 2022). Root exudates were determined by Sugar Pharma Technology Co., Ltd. (Beijing).

### Soil DNA extraction and sequencing

Genomic DNA was extracted from a 0.25 g soil sample using a TIANamp Soil DNA Kit (Tiangen Biotech, Beijing, China) following the manufacturer’s instructions. The quality and quantity of DNA were determined by the A260/280 ratio using a NanoDrop device (NanoDrop 2000, Germany) and electrophoresis (1% agarose gel, including a 1 kb plus ladder). The DNA samples from the soils of the same plot were pooled and stored at −80°C until PCR amplification. The V3–V4 hypervariable regions of bacterial 16S rRNA were amplified using the barcoded primers, B341F (CCACGGGNGGCWGCAG) and B785R (GACACHVGGGATCAATCC), and the fungal ITS2 regions were amplified using the barcoded primers, ITS3 (GATGAAGAACGYAGYRAA) and ITS4 (TCCTCCGCTATTGAATGC) (Toju et al., 2012; Klindworth et al., 2013). PCR conditions: the reaction mix (20 μL) contained 0.5 μL of DNA sample, 4 μL of 5× Fast-Pfu buffer, 2 μL of 2.5 mM dNTPs, 0.4 μL of each primer (5 μM), and 0.4 μL of Fast-Pfu polymerase (TransGen Biotech, Beijing, China). PCR amplification included 30 cycles at 95°C for 30 s, 55°C for 30 s, and 72°C for 30 s (T100 Thermal Cycler, Bio-red, CA, United States). Three independent PCRs for each DNA sample were performed and the triplicate products were pooled to minimize the bias of PCR amplification. The amplicon products were purified using an AxyPrep PCR Clean-up Kit (Axygen Biosciences, CA, United States) and underwent agarose gel electrophoresis. The concentrations of the purified PCR products were determined with QuantiFluorTM-ST (Promega, WI, United States; Gu et al., 2016). Purified PCR amplicons were sequenced using the Illumina MiSeq platform (300 bp paired-end reads; OriGene Technology Co., Ltd. Beijing, China).

High-quality paired-end reads of 16S rRNA and ITS sequences were merged using the FLASH software.^1^ Subsequently, the Mothur software^2^ was employed to filter the sequences and remove barcodes (Magoč and Salzberg, 2011). Operational taxonomic units (OTUs) were then generated using the UPARSE pipeline, which was based on the merged sequences (Edgar, 2013). Sequences exhibiting a similarity of ≥97% were grouped into similar OTUs (Edgar, 2013). To acquire taxonomic information about these OTUs, representative sequences of each OTU were generated and then aligned against two specific databases: the SILVA database (v132) for 16S sequences and the UNITE database (dynamic release 28.06.2017) for ITS sequences (Quast et al., 2012; Kõljalg et al., 2013). This alignment was accomplished using the RDP classifier.^3^

Alpha-diversity indices, including Sobs, Chao, and Shannon, were calculated using Mothur v. 1.34.4 (Zhang et al., 2021). The functional profiles of bacteria were generated employing the Functional Annotation of Prokaryotic Taxa (FAPROTAX 1.2.5) (Louca et al., 2016). Trophic classification of fungi was performed using FungalTraits, which used all possible, probable, and highly Probable results (Põlme et al., 2013). Heatmap based on Pearson bivariate correlation analysis between the relative abundance of microbial communities at the phylum level and metabolites were generated in R using the “Hmisc,” “reshape 2,” and “pheatmap” packages (Lian et al., 2020).

The raw sequences were deposited in NCBI’s Sequence Read Archive (SRA) under BioProject PRJNA1077876.

### Statistical analysis

Physiological and biochemical traits of plant, soil microbial community alpha-diversity and composition, and root exudate data were submitted to one-way ANOVA followed by Duncan’s multiple range test. Differences at *p* < 0.05 were regarded as statistically significant (Zhang et al., 2021). Conditioned constrained principal coordinate analysis (CPCoA) based on Bray-Curtis distance was used to visualize the associations among soil microbial community parameters and root exudates and stacked bar charts were generated using ImageGP (http://www.ehbio.com/Cloud_Platform/front/; Chen et al., 2021). Nonparametric permutational multivariate ANOVA (PERMANOVA) was conducted in R (version 4.2) using the “vegan” package (Lian et al., 2020).

Root exudates that exhibited significant differences among the four treatments were identified using partial least squares discriminant analysis (PLS-DA) with a variable importance in the projection (VIP) score greater than 1 and a significance level of *p* < 0.05, which was conducted in R using “ropls” package. The annotation of root exudates was carried out using the Kyoto Encyclopedia of Genes and Genomes (KEGG) database.^4^ The hexose/pentose (C6/C5) ratio equals the sum of the relative abundance of hexose (C6) divided by the sum of the relative abundance of Pentose (C5).

## Results

### Differences in plant characteristics under intercropping system

**Figure 1:**
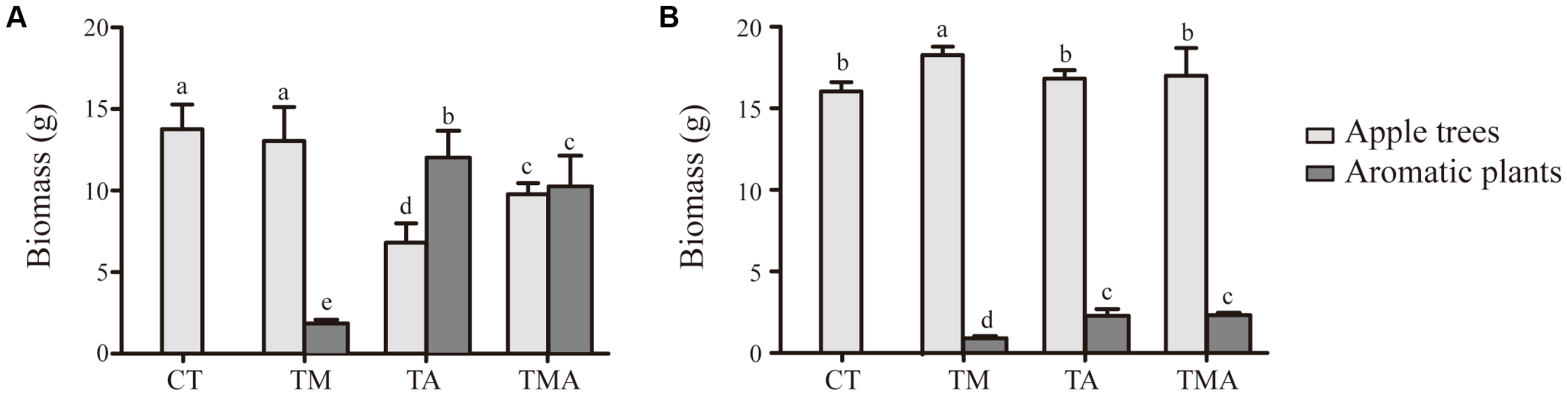
Changes in biomass of apple trees (*Malus hupehensis*) and aromatic plants in **(A)** above-ground and **(B)** under-ground between different intercropping treatments [Biomass, dry weight per pot (g/pot)]. Values are the mean ± SD (*n* = 3). Different letters indicate significant differences (*p* < 0.05) based on Duncan’s multiple range test. TM, Young apples intercropping with medium growth-potential *Mentha haplocalyx* Briq.; TA, Young apples intercropping with high growth-potential *Ageratum conyzoides* L.; TMA, as well as mixed intercropping with medium growth-potential *Mentha haplocalyx* Briq. and high growth-potential *Ageratum conyzoides* L.; CT, Only planted apple trees.

In the intercropping system, the above-ground and below-ground biomasses of the intercrops were quite similar between TA and TMA and notably higher than those in TM. Conversely, when it came to the host plant, we found that the above-ground biomass was reduced in TA and TMA compared to that in CT, while the below-ground biomass was increased in TMA, resulting in a higher root-to-shoot ratio (Figure 1).

Furthermore, in comparison to CT, TA led to increased levels of leaf and root MDA and P contents, while it decreased leaf chlorophyll a and b content, root SOD activity, and leaf and root CAT and POD activity, soluble protein, and N and K contents. In contrast, TM increased the leaf chlorophyll a and b contents; leaf CAT and POD activities; root soluble protein, MDA, N, and P contents; and root SOD activity, while decreasing leaf MDA content. Additionally, TMA increased root SOD activity and leaf and root MDA and P contents, while decreasing leaf and root soluble protein, N, and K contents and CAT and POD activities (Table 1). These findings indicate that the intercropping of aromatic plants inhibited the growth of apple trees, with TA exhibiting the most pronounced inhibition.

**Table 1:** Apple physiological indicators between intercropping treatment and control groups.

Indicator	CT	TM	TA	TMA
Chlorophyll (mg/kg)	3.96 ± 0.03 b	4.91 ± 0.07 a	3.85 ± 0.04 c	4.87 ± 0.05 a
Chla (mg/kg)	2.75 ± 0.09 c	3.52 ± 0.01 a	2.60 ± 0.04 d	3.32 ± 0.1 b
Chlb (mg/kg)	1.15 ± 0.11 c	1.32 ± 0.07 b	1.20 ± 0.07 bc	1.48 ± 0.05 a
Carotenoid content (mg/kg)	0.34 ± 0.05 b	0.41 ± 0.01 ab	0.38 ± 0.03 ab	0.46 ± 0.08 a
Soluble protein (mg/kg)	3.07 ± 0.05 a	3.06 ± 0.09 a	2.55 ± 0.05 c	2.85 ± 0.03 b
MDA (μmol/kg)	11.51 ± 0.24 c	10.19 ± 0.25 d	15.66 ± 0.18 a	13.44 ± 0.20 b
SOD (U/g)	45.28 ± 0.7 a	45.85 ± 0.71 a	45.62 ± 0.46 a	46.37 ± 1.17 a
CAT (U/g)	2578.78 ± 14.94 b	2770.79 ± 72.59 a	2182.95 ± 76.92 c	2121.37 ± 32.3 c
POD (U/g)	173.94 ± 4.38 b	188.66 ± 5.64 a	162.36 ± 2.84 c	98.97 ± 2.12 d
N (mg/g)	28.89 ± 1.59 a	29.51 ± 0.39 a	20.25 ± 1.25 c	25.27 ± 0.19 b
P (mg/g)	1.39 ± 0.05 c	1.31 ± 0.06 c	1.73 ± 0.05 a	1.56 ± 0.04 b
K (mg/g)	12.31 ± 0.44 a	12.82 ± 0.18 a	10.54 ± 0.35 b	11.10 ± 0.52 b
Soluble protein (mg/kg)	2.42 ± 0.07 b	2.55 ± 0.03 a	1.26 ± 0.04 d	1.79 ± 0.04 c
MDA (μmol/kg)	15.30 ± 0.39 d	18.79 ± 0.19 c	24.88 ± 0.65 a	20.02 ± 0.12 b
SOD (U/g)	66.96 ± 1.40 c	71.06 ± 1.75 b	63.61 ± 0.74 d	75.43 ± 1.54 a
CAT (U/g)	3234.21 ± 49.24 a	3266.44 ± 49.24 a	1504.28 ± 15.04 c	1713.45 ± 43.26 b
POD (U/g)	263.43 ± 6.90 a	274.06 ± 9.98 a	101.53 ± 2.07 c	192.33 ± 2.23 b
N (mg/g)	25.20 ± 1.28 b	34.47 ± 1.32 a	16.30 ± 0.41 d	19.48 ± 1.44 c
P (mg/g)	1.10 ± 0.01 c	1.88 ± 0.07 a	1.69 ± 0.08 b	1.94 ± 0.16 a
K (mg/g)	7.92 ± 0.25 b	9.75 ± 0.30 a	6.65 ± 0.42 c	8.09 ± 0.61 b

Values are the mean ± SD (*n* = 3). Different letters indicate significant differences (*p* < 0.05) based on Duncan’s multiple range test. Chlorophyll, The content of total chlorophyll (mg/kg); Chla, The content of chlorophyll a (mg/kg); Chlb, The content of chlorophyll b (mg/kg); Carotenoid content, The content of carotenoid (mg/kg); Soluble protein, The content of soluble protein per pot (mg/kg); MDA, Malondialdehyde content (μmol/kg); CAT, Catalase (U/g); POD, Peroxidase (U/g); N, Nitrogen (mg/g); P, Phosphorus (mg/g); K, Potassium (mg/g); TM, Young apples intercropping with medium growth-potential *Mentha haplocalyx* Briq.; TA, Young apples intercropping with high growth-potential *Ageratum conyzoides* L.; TMA, as well as mixed intercropping with medium growth-potential *Mentha haplocalyx* Briq. and high growth-potential *Ageratum conyzoides* L.; and CT, Tillage as a control.

### Differences in metabolite compositions in various treatments and root zones

**Figure 2:**
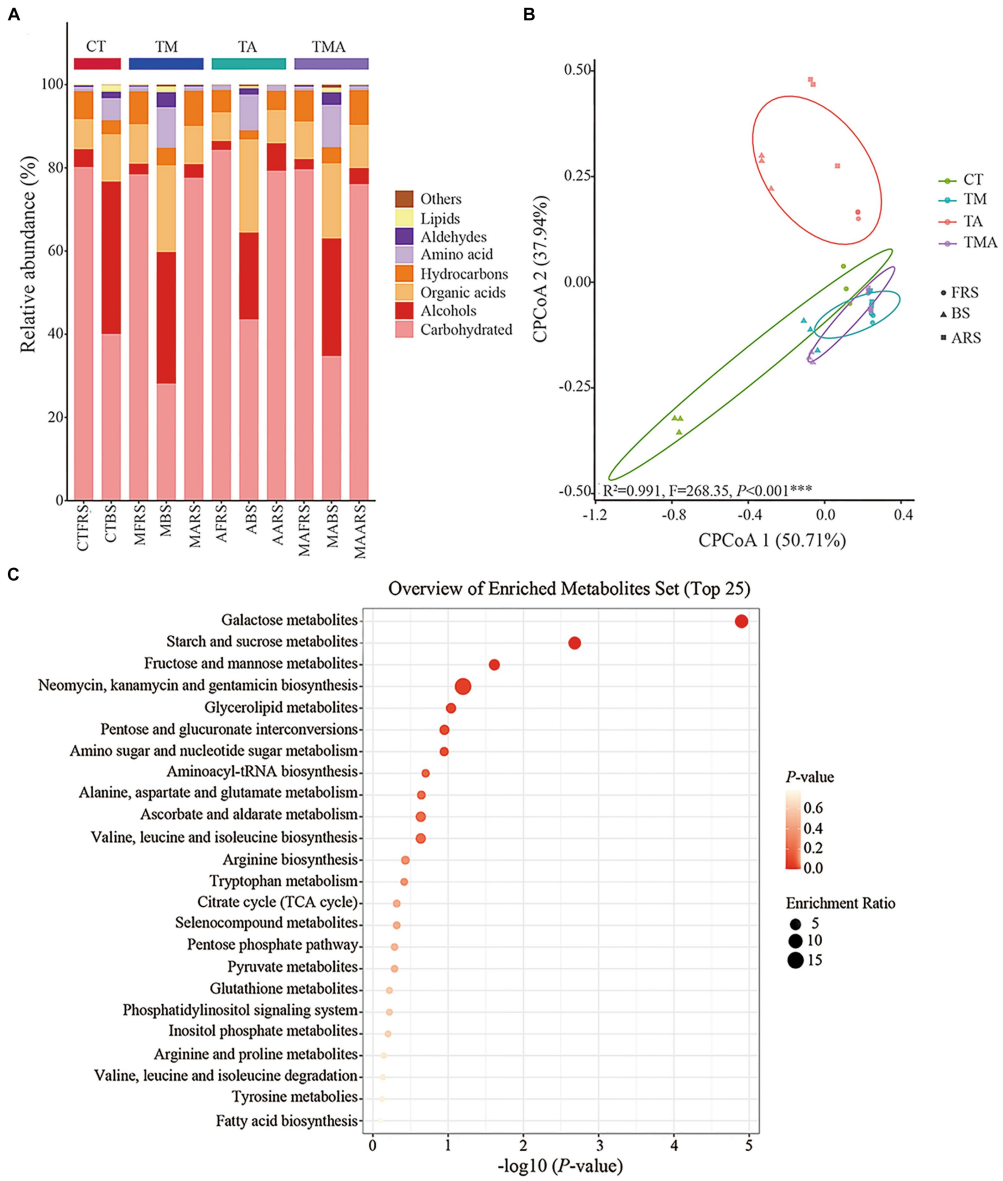
Compound and prediction of the metabolic pathway of root exudates in *Malus hupehensis* rhizosphere: **(A)** Stacked histogram of root exudates sorted by metaboanalyst; **(B)** CPCoA of root exudates in four intercropping treatments (CT, TM, TA, and TMA) and three ecological positions (FRS, BS, and ARS); and **(C)** analysis of the metabolic pathways of root exudates. TM, Young apples intercropping with medium growth-potential *Mentha haplocalyx* Briq.; TA, Young apples intercropping with high growth-potential *Ageratum conyzoides* L.; TMA, as well as mixed intercropping with medium growth-potential *Mentha haplocalyx* Briq. and high growth-potential *Ageratum conyzoides* L.; and CT, Only planted apple trees. FRS, Apples rhizosphere soil; BS, Relative to soil bulk; and ARS, Aromatic plant rhizosphere soil.

To identify the components of metabolites and their distribution in the different root zones, GC-MS was performed for the three soil phases in the four intercropping treatments. Overall, 72 compounds were identified and their relative abundance was calculated. Classification components included carbohydrates (26.63–84.77%), alcohols (2.11–35.27%), organic acids (6.95–21.09%), hydrocarbons (2.06–8.49%), amino acids (0.97–9.57%), aldehydes (0.022–3.40%), lipids (0.044–1.56%), others (0.030–0.69%), single component including trehalose (approximately 75.71%), galactose (approximately 25.67%), glycerol (approximately 23.20%), hexadecenoic acid (approximately 7.61%), and mannitol (approximately 4.78%) (Figure 2A). The alterations in soil metabolites revealed notable differences in the relative abundances of various carbon compounds, as illustrated by changes in the relative abundance of alcohols, organic acids, hydrocarbons, amino acids, and aldehydes across the four treatments and three soil phases (Figure 2A). In FRS, both TA and TMA exhibited an increase in carbohydrate content, accompanied by a decrease in the levels of organic acids and hydrocarbons, as compared to TM. However, it is worth noting that not all treatments involving aromatic plants had significantly different results compared to CT in this regard. In BS, TA was associated with increased levels of carbohydrates and organic acids, along with reduced levels of alcohol, hydrocarbons, amino acids, aldehydes, and lipids, when compared to TM and TMA, and most treatments involving aromatic plants were significantly different from CT. TA resulted in soil phase differences in the order FRS and ARS > BS for carbohydrates and hydrocarbons levels, BS > ARS > FRS for alcohol and organic acid levels, and BS > ARS and FRS for other components levels; TM resulted in soil phase differences in the order FRS and ARS > BS for carbohydrates and hydrocarbons levels and BS > ARS and FRS for other components levels; TMA resulted in soil phase differences in the order FRS > ARS > BS for carbohydrates, FRS and ARS > BS hydrocarbons levels, and BS > ARS and FRS for other components levels (Figure 2A). These effects were mainly caused by the different chemical compositions of the aromatic plant rhizospheric exudates, including lower levels of organic acids and hydrocarbons and higher levels of alcohols in TA (Figure 2A). CPCoA revealed significant sorting separation among the four treatments, with particularly noticeable distinctions among CT, TM, and TA. Furthermore, more pronounced sorting separations were observed within FRS, BS, and ARS (Figure 2B). These outcomes imply that intercropping with different species of aromatic plants and mixed intercropping induce substantial alterations in the composition of various soil phases within the root zones. Interestingly, the KEGG analysis highlighted that all soil metabolites were rich in galactose, starch, sucrose, fructose, and mannose metabolic pathways. This suggests that monosaccharides were dominant compounds within the soil metabolites of the intercropping system (Figure 2C).

**Figure 3:**
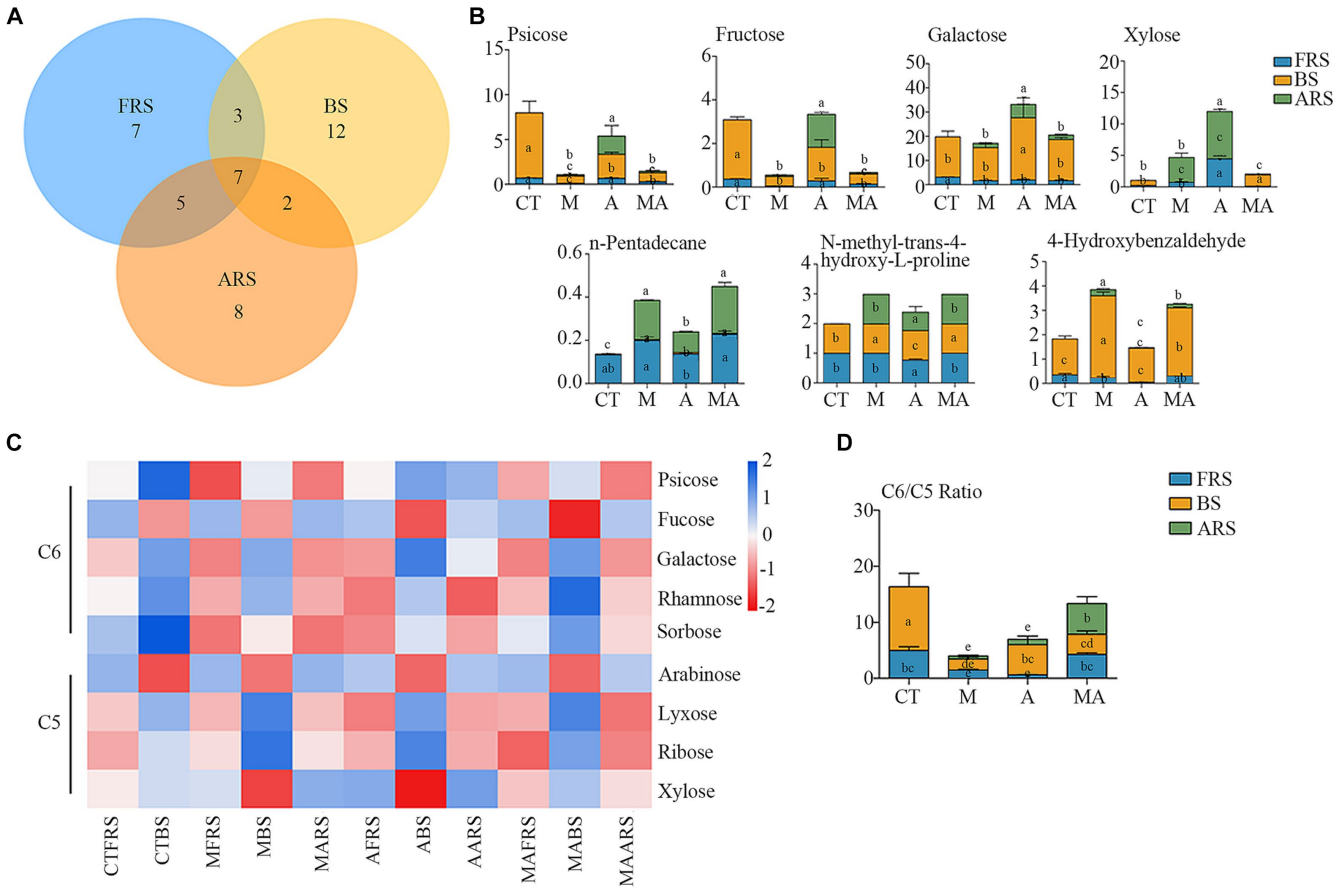
Changes of root exudation in content and carbon evaluation indicators among different intercropping treatments in three soil ecological niches: **(A)** Venn diagram of differential root exudates between intercropping treatments among FRS, BS, and ARS; **(B)** variation in content of seven core root exudates; **(C)** changes in the content of neutral sugars under treatment CT, TM, TA, and TMA; and **(D)** variation in content of hexose/pentose ratio of FRS, BS, and ARS.

In our analysis, of the 72 detected single compounds, 22 differential soil metabolites were identified in the rhizosphere soil (FRS), 24 in BS, and 20 in ARS. Additionally, a total of seven differential soil metabolites were detected across all three soil phases, as determined using PLS-DA analysis (VIP > 1 and *p* < 0.05) following intercropping (Figures 3A,B; Supplementary Figure S3). These multiple differences in single compounds among the root zones induced by various treatments inevitably led to changes in microbiome characteristics, thereby affecting soil carbon and nitrogen cycles and the nutrient supply to both intercrops and their host plants.

Among the carbohydrates that showed an increase because of intercropping with aromatic plants (Figures 3C,D; Supplementary Figure S2; Supplementary Table S1), TA notably increased the relative abundance of xylose (C5) and d-glucopyranose (C16), while reducing the relative abundance of arabinose (C5), fucose (C6), and glucose (C6) in FRS when compared with that in TM and TMA. This resulted in a lower C6/C5 ratio. In BS, TA increased the relative abundance of sucrose (C12) and galactose (C6), while reducing that of arabinose (C5) and rhamnose (C6), leading to a higher C6/C5 ratio compared with that in TM and TMA. This observed pattern could be attributed to the higher levels of sucrose (C12), glucose (C6), fructose (C6), d-glucopyranose (C16), and psicose (C6), as well as lower levels of trehalose (C12), xylose (C5), fucose (C6), and arabinose (C5) in ARS under TA compared to those under TM and TMA. These differences resulted in variations in three soil phases with trehalose (C12), fucose (C6), and xylose (C5) exhibiting FRS > ARS > BS, d-glucopyranose (C16) exhibiting ARS > FRS > BS, and sucrose (C12) showing BS > ARS > FRS. Additionally, two soil phase differences were observed for glucose (C6) with ARS > BS and FRS, fructose (C6) and psicose (C6) with ARS and BS > FRS, and other carbohydrate components with BS > FRS and ARS. Among these, TA resulted in soil phase differences with the order BS > ARS and FRS for the C6/C5 ratio.

### Soil microbial diversity in different treatments and root zones

**Figure 4:**
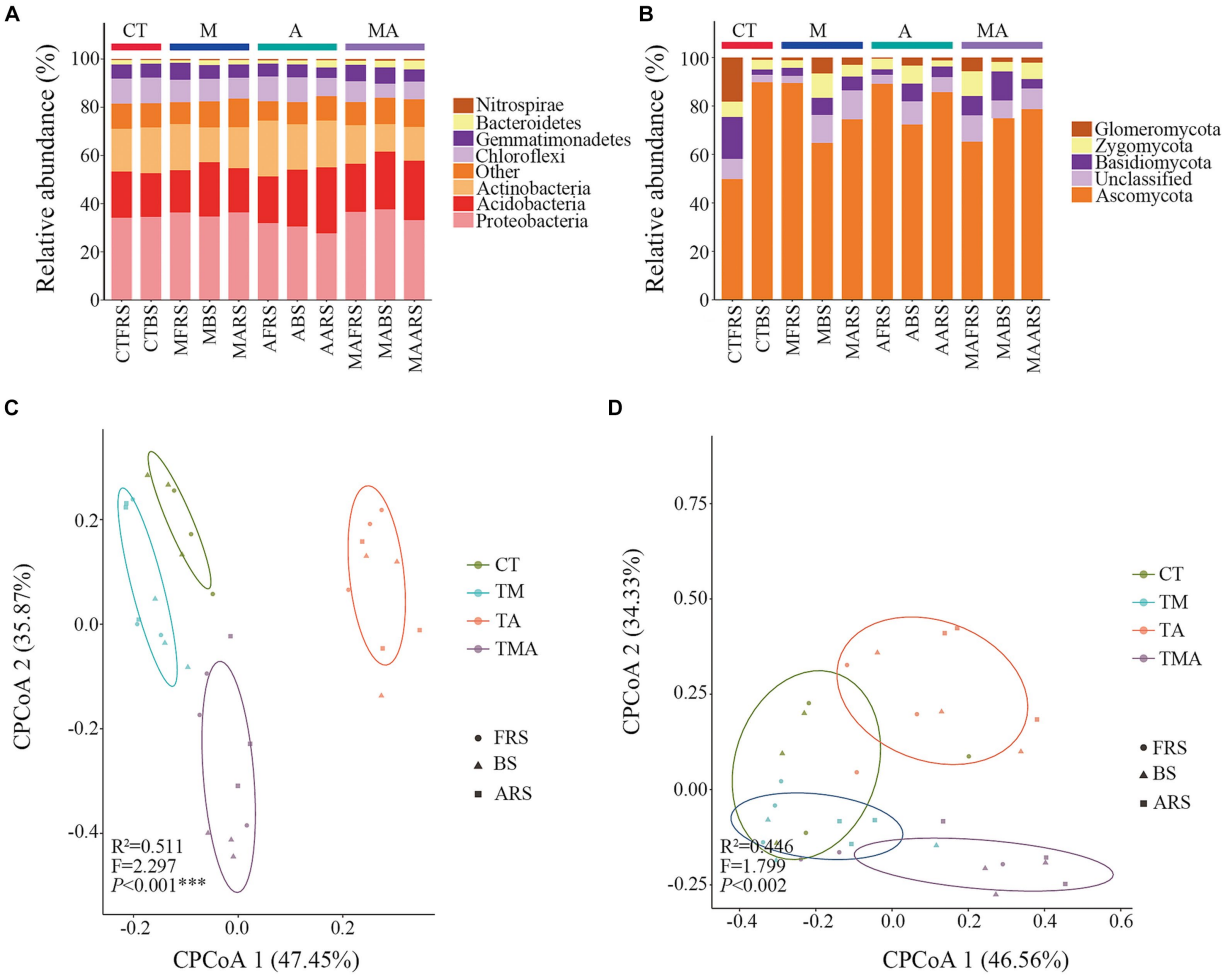
Phylum composition and beta diversity of the communities in soil with different intercropped aromatic plants: **(A)** bacteria communities at the phylum level; **(B)** fungi communities at the phylum level; **(C)** beta diversity of bacterial communities; and **(D)** beta diversity of fungal communities. cPCoA shows the structures of the soil bacterial and fungal communities. TM, Young apples intercropping with medium growth-potential *Mentha haplocalyx* Briq.; TA, Young apples intercropping with high growth-potential *Ageratum conyzoides* L.; TMA, as well as mixed intercropping with medium growth-potential *Mentha haplocalyx* Briq. and high growth-potential *Ageratum conyzoides* L.; and CT, Only planted apple trees. FRS, Apples rhizosphere soil; BS, Relative to soil bulk; and ARS, Aromatic plant rhizosphere soil. The relative abundance of bacterial and fungi community <1% was filtrated in Phylum composition.

In our analysis, 2,102,831 high-quality bacterial sequences and 2,213,651 high-quality fungal sequences were obtained from all the samples. These sequences were grouped into 16,991 and 3,267 OTUs, respectively, using a 97% sequence similarity cut-off. The dominant bacterial phyla in the total bacterial community were Proteobacteria (approximately 37.65%), Acidobacteria (approximately 27.35%), Actinobacteria (approximately 23.19%), Chloroflexi (approximately 10.68%), Gemmatimonadetes (7.02%), Bacteroidetes (approximately 3.68%), and Nitrospirae (approximately 0.70%; Figure 4A). On the other hand, the dominant fungal phyla in the total fungal community included Ascomycota (approximately 89.77%), Glomeromycota (approximately 18.21%), Basidiomycota (approximately 17.27%), and Zygomycota (approximately 39.88%; Figure 4B).

Comparing these communities with those in CT, we observed that intercropping had various effects on alpha diversity. For the bacterial community, intercropping reduced alpha diversity in FRS, while TA and TMA reduced the Sob, Chao, and Shannon indices in BS, primarily due to the lower alpha diversity observed in TA compared to that in TM and TMA in ARS. Conversely, for the fungal community, intercropping increased alpha diversity in FRS. In BS, TMA reduced the Chaos, while TA and TMA increased the Shannon index. These effects were attributed to differences in alpha diversity among the treatments in FRS and BS, with varying impacts on different aspects of diversity (Supplementary Table S2).

We employed CPCoA to assess variations in microbial communities across different intercropping treatments at the OTU level. The CPCoA plots depicted differences in bacterial and fungal communities across the intercropping treatments in FRS, BS, and ARS. The larger distances observed between each treatment along CPCoA1 and CPCoA2 indicated that intercropping with aromatic plants and their mixtures had a more pronounced impact on the community structure of bacteria compared to that of fungi (Figures 4C,D).

### Soil microbial composition in the different treatments and root zones

When compared to the CT, intercropping with aromatic plants had significant effects on the relative abundance of dominant bacteria at the taxon level (Figures 4A,B; Supplementary Figure S4). In FRS, TA led to an increase in the relative abundance of Actinobacteria, Nocardioidaceae, *Nocardioides*, and *Arthrobacter*, while reducing the relative abundance of Deltaproteobacteria, Myxococcales, *Haliangiaceae*, and *Halobacterium*. Additionally, the relative abundances of Proteobacteria, Gammaproteobacteria, *Gemmatimonas*, Acidobacteriales, Acidobacteriaceae, and *Candidatus Koribacter* were higher in TM and TMA than in TA, while those of Thermomicrobia and *Acidimicrobiales* were lower.

In BS, TA promoted the relative abundances of Acidobacteria, Holophagae, Subgroup-4, Nitrospirae, Xanthomonadales, *Arthrobacter*, and Claroideoglomeraceae, while reducing those of Deltaproteobacteria, Myxococcales, Rhodospirillaceae, and *Dongia* (Figures 4A,B; Supplementary Figure S4). These differences in bacterial taxa can be attributed to variations in the relative abundance of rhizospheric microbes induced by intercropping with aromatic plants (Figures 4A,B; Supplementary Figure S4). Notably, the relative abundances of Alphaproteobacteria, Deltaproteobacteria, Rhodospirillales, Myxococcales, Haliangiaceae, and *Halobacterium* were higher in TM and TMA compared to those in TA. Conversely, the relative abundances of *Acidimicrobiia*, Nocardioidaceae, *Nocardioides*, *Arthrobacter*, Eurotiomycetes, Eurotiales, Trichocomaceae, and *Myrothecium* were lower in TM and TMA than in TA. Furthermore, the relative abundances of Bacteroidetes, Dongia, Proteobacteria, Dothideomycetes, and Cantharellales were higher in TMA compared to in TA, while that of *Actinobacteria* was lower. Additionally, TM also exhibited higher relative abundances of Proteobacteria, Betaproteobacteria, and Dongia than TA and lower abundances of RB41 and Xanthomonadaceae.

Comparisons among FRS, BS, and ARS also revealed significant differences in the relative microbial abundance resulting from intercropping with aromatic plants (Figures 4A,B; Supplementary Figure S4). In TA, microbial taxa with higher abundances in FRS than in BS and/or ARS included Actinobacteria, Rhodospirillales, Nocardioidaceae, *Nocardioides*, *Arthrobacter*, and Dothideomycetes; those with higher abundances in BS than in FRS and/or ARS included Proteobacteria, Mortierellaceae, Claroideoglomeraceae, and *Mortierella*. Conversely, those with higher abundances in ARS than in FRS and BS included Acidobacteria, Sphingobacteriia, Subgroup-6, Subgroup-4, Eurotiomycetes, Eurotiales, and Trichocomaceae. Under TA, eutrophic bacteria, including Actinobacteria, Nocardiaceae and genera, *Arthrobacter* and Dothideomycetes in FRS > BS and ARS; the oligotrophic bacteria Acidobacteria, Subgroup-6, Subgroup-4, Sphingobacteria (Bacteroidetes), and Eurotiomycetes, Zygomycetes in ARS > BS and FRS (Figure 4A; Supplementary Figure S4).

### Carbon and nitrogen cycle-related soil microbial function differences in the various treatments and root zones

**Figure 5:**
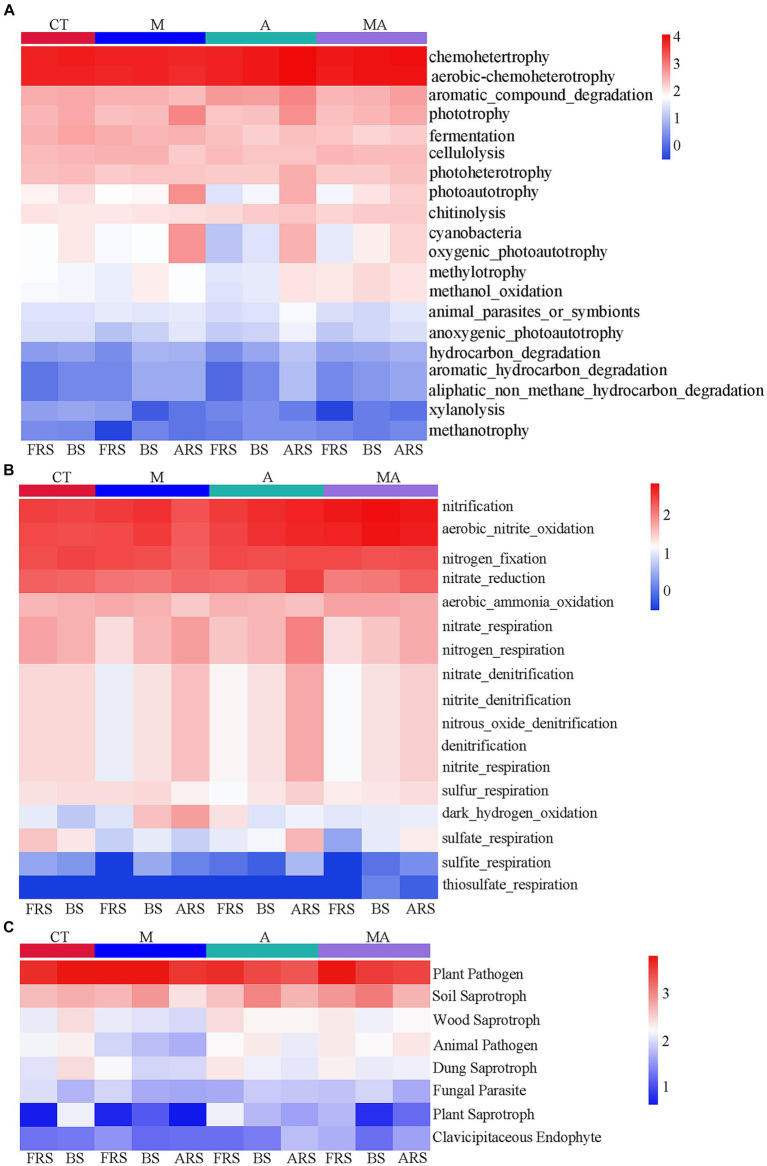
Heatmap of prediction of functional groups in the soil of bacterial and fungal communities based on OTUs from 16S rRNA and ITS sequencing: **(A)** C-cycle functional prediction of bacterial communities; **(B)** N-cycle functional prediction of bacterial communities; and **(C)** ecological functions of fungal communities. TM, Young apples intercropping with medium growth-potential *Mentha haplocalyx* Briq.; TA, Young apples intercropping with high growth-potential *Ageratum conyzoides* L.; TMA, as well as mixed intercropping with medium growth-potential *Mentha haplocalyx* Briq. and high growth-potential *Ageratum conyzoides* L.; and CT, Only planted apple trees. FRS, Apples rhizosphere soil; BS, Relative to soil bulk; and ARS, Aromatic plant rhizosphere soil.

Based on the FAPROTAX database, we observed that many carbon and nitrogen cycle-related functions exhibited significant differences in FRS, BS, and ARS because of the differences in the microbial functions in the rhizosphere of aromatic plants (Figure 5). Certain carbon-cycle-related functions exhibited higher levels in ARS than in both FRS and BS, which included phototrophy, photoautotrophy, anoxygenic-photoautotrophy, and cyanobacteria across all intercropping treatments. In addition, TA and TMA showed increased levels of chemoheterotrophy, aerobic-chemoheterotrophy, aromatic-compound degradation, photoheterotrophy, animal-parasite or symbionts, hydrocarbon degradation, aromatic-hydrocarbon degradation, and aliphatic-non-methane-hydrocarbon degradation. A similar pattern was observed in the nitrogen cycle, with functions including nitrate reduction, nitrate-respiration, nitrogen-respiration, nitrate-denitrification, nitrite-denitrification, nitrous-oxide, denitrification, and sulfite respiration exhibiting higher levels in ARS than in FRS and BS. However, it is worth noting that fungal-related functions did not display significant changes across the different soil phases. This suggests that alterations in rhizospheric carbon- and nitrogen-cycle-related functions were primarily driven by the intercropping treatments, particularly TA and TMA, while fungal functions remained relatively stable across the soil phases.

### Correlation between soil microbial communities and soil metabolites

**Figure 6:**
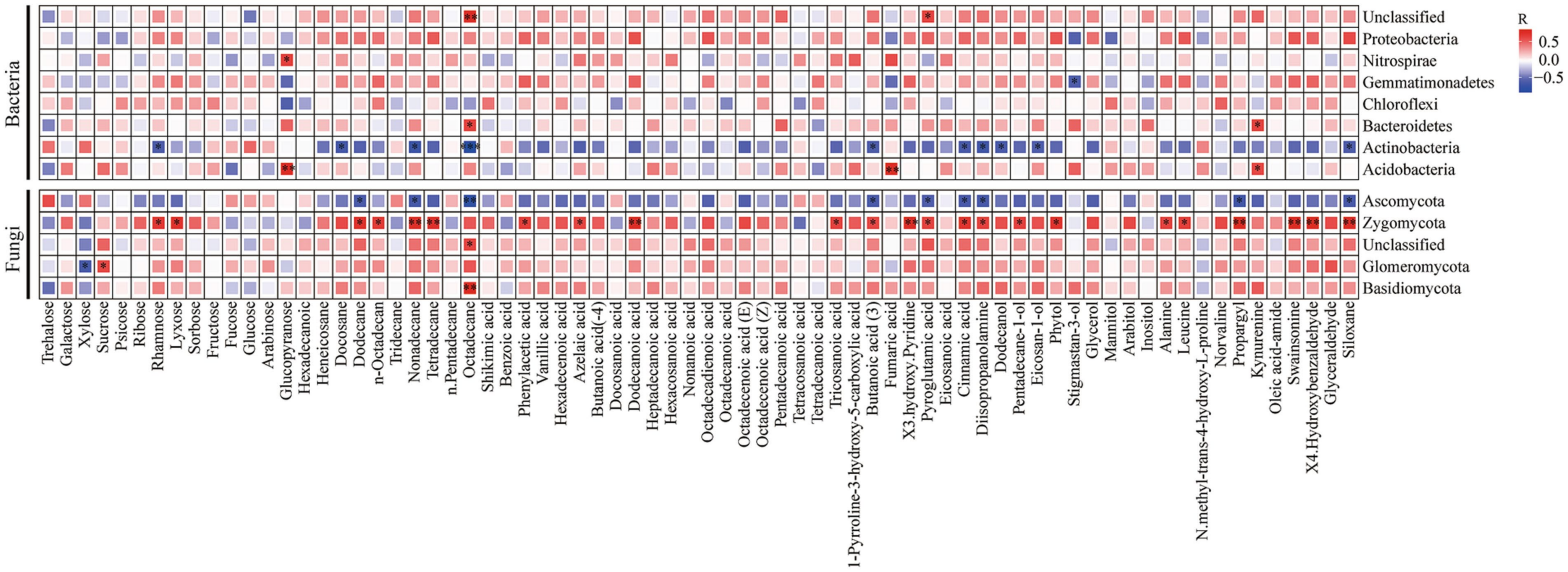
Pearson’s correlation analysis between soil microbes at phylum level (the relative abundance >1%) and soil metabolites. Red boxes represent positive correlations, while blue boxes represent negative correlations. Black asterisks indicate statistical significance: ^*^p < 0.05; ^**^p < 0.01, and ^***^p < 0.001.

The correlations between soil microbial communities and soil metabolites were obtained through Pearson’s correlation analysis (Figure 6). Proteobacteria were positively correlated with carbohydrates and negatively correlated with organic acids, amino acids, and aldehydes. Gemmatimonadetes were positively correlated with carbohydrates, and negatively correlated with amino acids and aldehydes. Actinobacteria were positively correlated with amino acids and negatively correlated with hydrocarbons.

## Discussion

### Resource competition between intercrops and host plants led to the reduced growth of young host plants

Antagonism is a pervasive and detrimental phenomenon commonly observed in intercropping systems. It significantly impedes the growth, development, biological yield, and economic value of the host plant. In agroforestry intercropping systems, particularly in forestlands and orchards, a crucial strategy for effective soil management involves the careful selection of intercrop species to avoid adverse effects on perennial forests and fruit trees, as emphasized in previous research (Castellano-Hinojosa and Strauss, 2020). However, despite its importance, the underlying mechanisms responsible for the antagonistic effects of various intercrops on different host plants within such systems have remained elusive. This knowledge gap has limited the accurate selection of intercrops that can coexist harmoniously with the main crop. In our study, we investigated the impact of intercropping with *Ageratum conyzoides* (TA) on the growth dynamics of both the host plant and *A. conyzoides*. We observed a decrease in the above-ground biomass of the host plant and a simultaneous increase in both the above-ground and below-ground biomass of *A. conyzoides* under TA treatment. Consequently, the host plant exhibited a higher root-to-shoot ratio while *A. conyzoides* exhibited a more balanced root-to-shoot ratio. Moreover, TA led to a reduction in chlorophyll content of the leaves and antioxidant activity, as well as N and K levels in both the leaves and roots of the apple tree, in contrast to CT and TM. These findings suggest the existence of mutual inhibition between the apple tree and *A. conyzoides*. This inhibition manifested as a reduced distribution of above-ground biomass in the host plant, contributing to the higher root-to-shoot ratio observed. These effects may be attributed to the inadequate nutrient supply in the rhizosphere soil of the apple tree caused by the presence of *A. conyzoides*. The low assimilative capacity and antioxidant activity of the plants further restricted the growth of the apple tree, primarily owing to indirect resource competition. Notably, chemical interference in soil chemical properties and nutrient cycling may have played a pivotal role in mediating these observed effects (Figure 1; Table 1; Cappelli et al., 2022).

### The discrepancy in chemical distribution patterns of rhizosphere exudates between intercrops and host plants led to the reduced growth of young host plants

The relationship between root exudates from host plants and intercrops is widely recognized as mutually beneficial in intercropping systems (Zhang et al., 2021). Nevertheless, a comprehensive investigation into the intricate chemical composition of root exudates from a diverse range of plant species, especially in woody and herbal plant intercropping systems has been lacking (Koprivova and Kopriva, 2022). It is understood that low-molecular-weight compounds such as amino acids, organic acids, sugars, phenolics, and various other secondary root exudates constitute the bulk of root exudates (Bais et al., 2006). Through these chemical signals, host plants and intercrops engage in ongoing communication, rapidly detecting and responding to each other’s presence, and adapting their strategies to gain an advantage in resource competition (Callaway and Aschehoug, 2000). Our study yielded insights into how intercropping with aromatic plants can significantly modify the composition and content of metabolites across three distinct soil phases (Figures 2A,B). Specifically, TA led to a reduction in the content of alcohols, aldehydes, and hydrocarbons when compared to CT. Additionally, TA exhibited lower levels of alcohols and aldehydes when compared to TM and TMA in FRS. Furthermore, in BS, TA displayed an increase in organic acid and amino acid content and a decrease in alcohol and lipid content relative to CT. Similarly, TA showed higher levels of carbohydrates and amino acids and lower levels of alcohols, aldehydes, amino acids, hydrocarbons, and lipids than TM and TMA. Notably, in ARS, TA exhibited lower levels of organic acids, aldehydes, and higher alcohols than TM and TMA. These findings collectively suggest that intercropping with *A. conyzoides* induced significant alterations in the chemical composition of soil metabolites, particularly in FRS and BS, which had negative implications for the allocation of dry matter in the host plant. Organic acids, which are considered secondary metabolites, play crucial roles in regulating pH, allelopathic interactions, defense mechanisms, and osmotic balance, as well as nutrient decomposition within plant root zones. Alcohols, amino acids, and aldehydes are involved in regulating biological signals, osmotic balance, and nutrient transformations within the root zone. However, the intricate relationships among soil metabolites in these processes remain complex and require further investigation. An analysis of the KEGG pathways revealed that soil metabolites were primarily associated with galactose metabolism, starch and sucrose metabolic pathways, and fructose and mannose metabolic pathways. In these pathways, the composition and proportion of carbohydrates emerged as the key distinguishing features among the intercropping treatments and soil phases.

Soil-neutral sugars play a pivotal role as both energy sources and osmotic regulators, influencing soil aggregation, transformation of organic carbon, water retention, and conversion into plant-available nutrients. These processes are primarily driven by microbes (Gunina and Kuzyakov, 2015). In the context of our study, it is important to distinguish between pentoses (such as arabinose, lyxose, ribose, and xylose) derived from plant exudates and deposition, and hexoses (including psicose, fucose, galactose, rhamnose, and sorbose) originating from microbial sources and their decomposition. The ratio of C6/C5 serves as a valuable indicator, reflecting differences in soil treatments, rhizosphere secretion processes, and various factors affecting microbial degradation and transformation in different soil types (Bischoff et al., 2018). In our study, compared with TM and TMA, TA increased pentose and decreased hexose in FRS and increased hexose and decreased pentose in BS, which resulted in decreased C6/C5 in FRS and increased C6/C5 in BS (Figure 3D), primarily due to the higher hexose levels and lower pentose levels in ARS. It can be speculated that the higher C6/C5 ratio observed in ARS had a cascading effect on BS in TA, leading to a higher C6/C5 ratio owing to chemical transduction. In contrast, fruit trees typically decreased C6/C5 ratios in their rhizosphere soils. This is attributed to the reduction of hexose levels and the allocation of carbon by fruit trees to their root systems, which may involve sacrificing above-ground carbon input. Consequently, this strategy results in a higher R/S ratio. To summarize, our hypothesis posits that in the case of TA, the elevated C6/C5 ratio in ARS influences a higher C6/C5 ratio in BS while causing a lower C6/C5 ratio in FRS. This discrepancy in carbohydrate distribution patterns between FRS and BS prompts the root system of apple trees to regulate the balance of their own secreted neutral sugars and microbial degradation. In doing so, it allocates more carbon to the root system, actively competing for nutrients at the distal end of the root.

### Microbial redistribution between intercrops and host plants reduced the growth of young host plants

Root exudates play a pivotal role in mediating interactions between roots and microbes in the soil (Neal et al., 2012). In intercropping systems, intercrops and host plants actively or passively secrete root exudates to adapt to their specific soil environments, gaining advantages in resource competition (Zhang et al., 2021). Microbes, serving as decomposers of soil organic matter, engage in intricate communication with host roots through mechanisms such as chemotaxis (in response to C-rich environments), chemical signaling, and motility traits to compete for resources. This microbial interaction produces signals that initiate colonization and functional associations, influencing the nature of plant–microbe interactions—whether they are synergistic or antagonistic (Bais et al., 2006). Therefore, the associations established between the roots and microbes can determine the overall dynamics of intercrop and host-crop interactions. Our study revealed that TA led to a decrease in alpha diversity within the bacterial community in both FRS and BS compared to CT. In contrast, TA increased the alpha diversity within the fungal community in FRS and BS relative to CT. However, TA decreased the alpha diversity of both bacterial and fungal communities in ARS compared to TM and TMA (Supplementary Table S1). These findings suggest that TA enriched specific bacterial populations in FRS and ARS, enabling them to better compete for nutrients. By altering the composition of the inter-root microbial diversity of fruit trees and hindering the optimization of microbial diversity, fruit trees under TA may favor their root systems, utilize their carbon resources, and employ chemical interventions in root secretions to suppress the expansion of *A. houstonianum* (Xu et al., 2021).

Plant–microbe interactions mediated by root exudates in the rhizosphere are critical for a range of intrinsic processes, including carbon sequestration, ecosystem functioning, and nutrient cycling (Singh et al., 2004). In our study, there was a significant correlation between bacterial communities and soil metabolites, rather than fungal communities. The composition and quantity of specific microbes in the soil significantly impact a plant’s ability to obtain nutrients (Gschwendtner et al., 2011) Our results indicate that TA led to a higher relative abundance of taxon members of Actinobacteria (including Actinobacteria, Nocardioidaceae, *Nocardioides*, and *Arthrobacter*), Nitrospirae (*Nitrospira*), and Xanthomonadaceae, while reducing the abundance of Proteobacteria compared to TM in FRS. Furthermore, these microbial abundances were higher in TA than in CT (Figures 4A,B; Supplementary Figure S4). Simultaneously, TA resulted in higher relative abundances of Thermomicrobia, Acidimicrobiales, Hyphomicrobiaceae, and Zygomycetes and lower relative abundances of Rhodospirillales, Gemmatimonadetes, and Acidobacteriales compared to TM in FRS; there were no significant differences when compared with those in CT (Figures 4A,B; Supplementary Figure S4). These findings suggest that the composition of the bacterial community in FRS shifted from a diverse range of dominant bacteria to a smaller number of dominant species under the influence of TA. The abundance of many dominant bacterial members decreased, and the proportion of members from different classes was altered by TA. These changes in bacterial abundance may be due to the preference of these microbes for the low hexose/pentose ratio in FRS or the mild allelopathic effects of lower phenolic acids in the rhizosphere of fruit trees (Koranda et al., 2014). The altered proportions of Actinobacteria, which are involved in cellulose, lignin, and pectin decomposition, and Nitrosospira, engaged in the nitrite environment, play crucial roles in carbon and nitrogen cycling in natural ecosystems. They enhance soil nutrient availability, making it easier for crops to directly absorb and utilize nutrients (Eisenlord et al., 2012). The decline in the relative abundance of Gemmatimonadetes may reflect the interaction between these nutrient-rich symbiotic bacteria and their preference for specific soil moisture conditions within soil aggregates in FRS (Fitzpatrick et al., 2018). The balance between Actinobacteria and Proteobacteria in the bacterial community composition might be adjusted to cope with competition between intercrops and host plants.

Many dominant members of the bacterial community participate in processes such as organic material decomposition, carbon and nitrogen fixation, soil carbon and nitrogen cycling, and carbon sequestration. Our results indicated that under TA, the chemical composition of soil metabolites in BS arising from interactions between intercrops and host plants influenced the composition and structure of Proteobacteria and Actinobacteria. These findings highlight the community’s adaptability to varying environmental factors, enabling them to balance nutrient and carbon cycle (Lunsmann et al., 2016), thereby enhancing carbon storage in the soil.

The changes in the eutrophic/oligotrophic ratio are related to the selective effects of root exudates on soil microbial communities (Cappelli et al., 2022; Chisholm et al., 2023). Under TA, eutrophic bacteria were enriched in FRS (FRS > ARS and BS), while oligotrophic bacteria were enriched in ARS (ARS > FRS and BS; Figures 4A,B; Supplementary Figure S4). These results suggested that competing parties assemble specific microbial functional groups through the chemical composition of their secretions and thus actively and passively develop dominance in nutrient catabolism and acquisition (Fitzpatrick et al., 2018). We conducted a comprehensive analysis of microbial community functions related to the soil carbon and nitrogen cycles in response to intercropping and our findings revealed several significant changes. Under the influence of TA, there was an increase in the relative abundance of bacterial aromatic-compound degradation functions associated with the carbon cycle in FRS. In BS, TA led to an increase in functions related to nitrification and aerobic nitrite oxidation, which are vital components of the nitrogen cycle, compared to TM. These shifts in microbial community functions were attributed to a higher abundance of functions such as chemoheterotrophy, aerobic-chemoheterotrophy, aromatic-compound degradation, photoheterotrophy, and nitrate reduction and lower relative abundances of phototrophy, cyanobacteria, and photoautotrophy in ARS (Figure 5). These findings indicated that intercropping with aromatic plants, particularly TA, significantly impacts soil carbon and nitrogen cycles. ARS exhibited higher carbon and nitrogen cycle functions than FRS and BS under TA conditions, underscoring the intricate interactions and effects of intercropping on soil ecosystem processes. This suggests that *A. conyzoides* has a more dominant carbon and nitrogen-cycle-related microbial functional group that performs organic matter decomposition in their rhizosphere, which also forces apple trees to expand their root systems to passively adapt to this competitive pressure (Bischoff et al., 2018).

We must point out that the changes in soil metabolites in this study are not equivalent to the metabolites of root exudates; the contribution of metabolites produced by native soil microbial communities cannot be ignored (Lian et al., 2020). Intercropping inevitably affects the root exudates of the host plant and then affects the metabolic activity of the composition of the soil microbial community, which affects soil microbial extracellular metabolites (Zhalnina et al., 2018). Thus, alterations in soil metabolite may be due in part to extracellular compounds released by the soil microbial community. The impact of root exudates on soil microbial community needs to be further explored. Moreover, the function of soil microbial taxa was predicted based on the FAPROTAX database and FungalTraits. Thus, the function of soil microbial taxa needs to isolate representative microbes to verify in the future.

## Conclusion

TA decreased the above-ground biomass of apple trees and had higher above-ground biomass of intercrops than CT, TM, and TMA, suggesting that the interspecific competition was the most pronounced in intercropping in TA. TA reshaped the soil microbial community, increasing taxon members of Actinobacteria and reducing Proteobacteria in FRS. In addition, TA reduced organic acids, alcohols, carbohydrates, and hydrocarbons in FRS, increased the pentose content, and decreased the C6/C5 ratio. These impacts led to a higher eutrophic/oligotrophic bacteria ratio in TA. This study provides a novel perspective on how interspecific competition, mediated by root exudates and microbial community feedback, influences plant growth and development.

## Data availability statement

The data presented in this study has been deposited in the NCBI repository, accession number PRJNA10806876, at URL: http://www.ncbi.nlm.nih.gov/bioproject/1080687.

## Author contributions

MZ: Data curation, Methodology, Visualization, Writing – original draft. YS: Data curation, Methodology, Visualization, Writing – review & editing. MD: Methodology, Visualization, Writing – review & editing. KZ: Data curation, Writing – original draft. JZ: Data curation, Writing – original draft. XQ: Conceptualization, Data curation, Funding acquisition, Supervision, Writing – review & editing. YY: Data curation, Writing – review & editing.

## Footnotes

^1^ http://ccb.jhu.edu/software/FLASH/index.shtml

^2^ https://mothur.org

^3^ https://rdp.cme.msu.edu/

^4^ https://www.kegg.jp/
