# Supplementary material for: Hexose/pentose ratio in rhizosphere exudates-mediated soil eutrophic/oligotrophic bacteria regulates the growth pattern of host plant in young apple–aromatic plant intercropping systems
Source: Front Microbiol. 2024 Mar 25;15:1364355. doi: 10.3389/fmicb.2024.1364355 (PMC11000693; doi:10.3389/fmicb.2024.1364355)
Supplement: Supplementary file 2 [file Data_Sheet_1.docx]

Hexose/pentose ratio in rhizosphere exudates mediated soil eutrophic/oligotrophic bacteria regulates the growth pattern of host plant in young apple–aromatic plant intercropping system


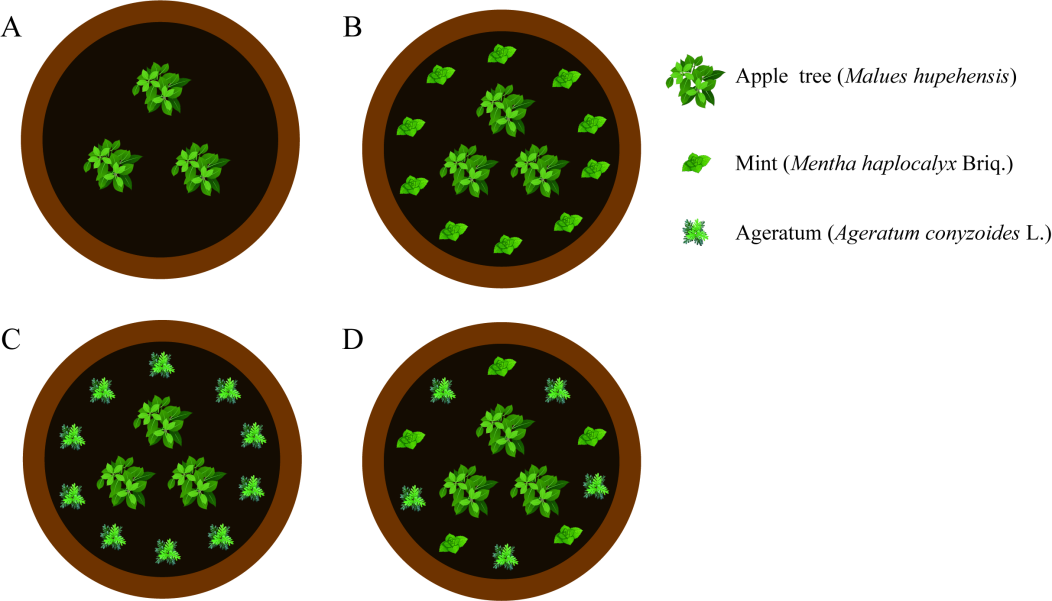


**Supplementary Figure 1.** Experimental design diagram. (A) CT; (B) TM; (C) TA; (D) TMA.


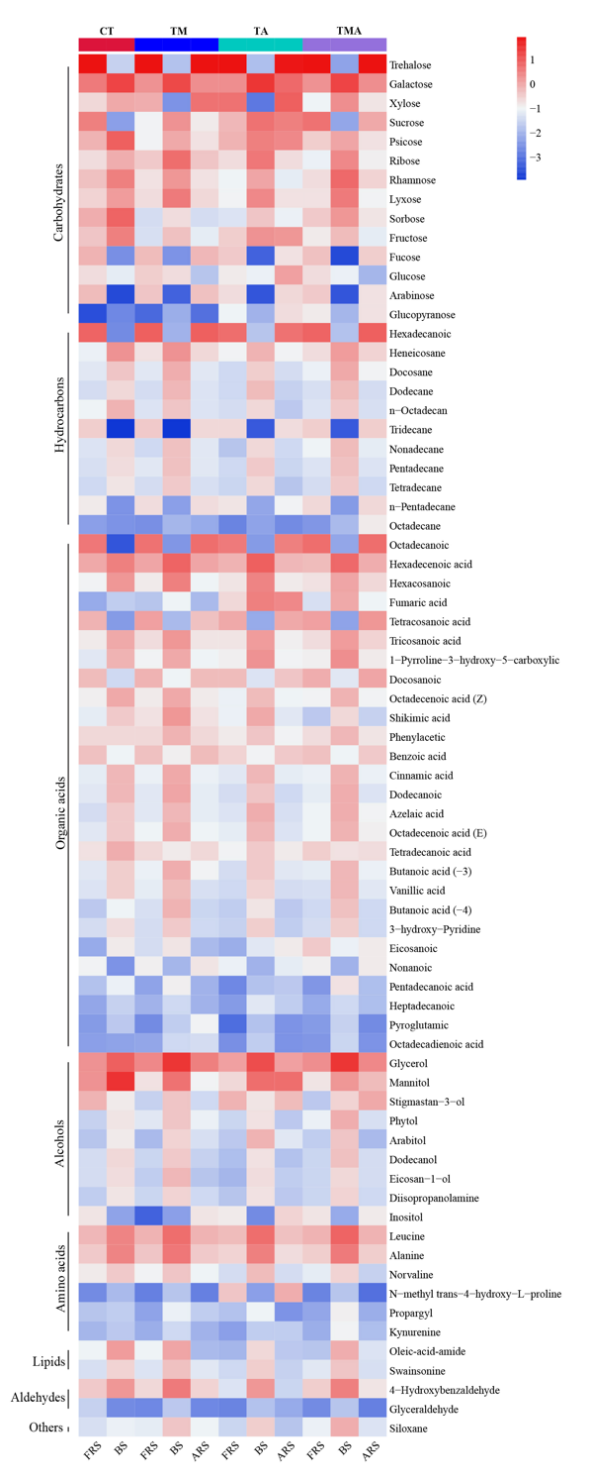


**Supplementary Figure 2.** Differences of root exudates in intercropping groups and soil ecological niche.

Notes: TM, young apples intercropping with medium growth-potential *Mentha haplocalyx* Briq.; TA, young apples intercropping with high growth-potential *Ageratum conyzoides* L.; TMA, as well as mixed intercropping with medium growth-potential *Mentha haplocalyx* Briq. and high growth-potential *Ageratum conyzoides* L.; CT, only planted apple trees. FRS, apples rhizosphere soil; BS, relative to soil bulk; ARS, aromatic plant rhizosphere soil.

.


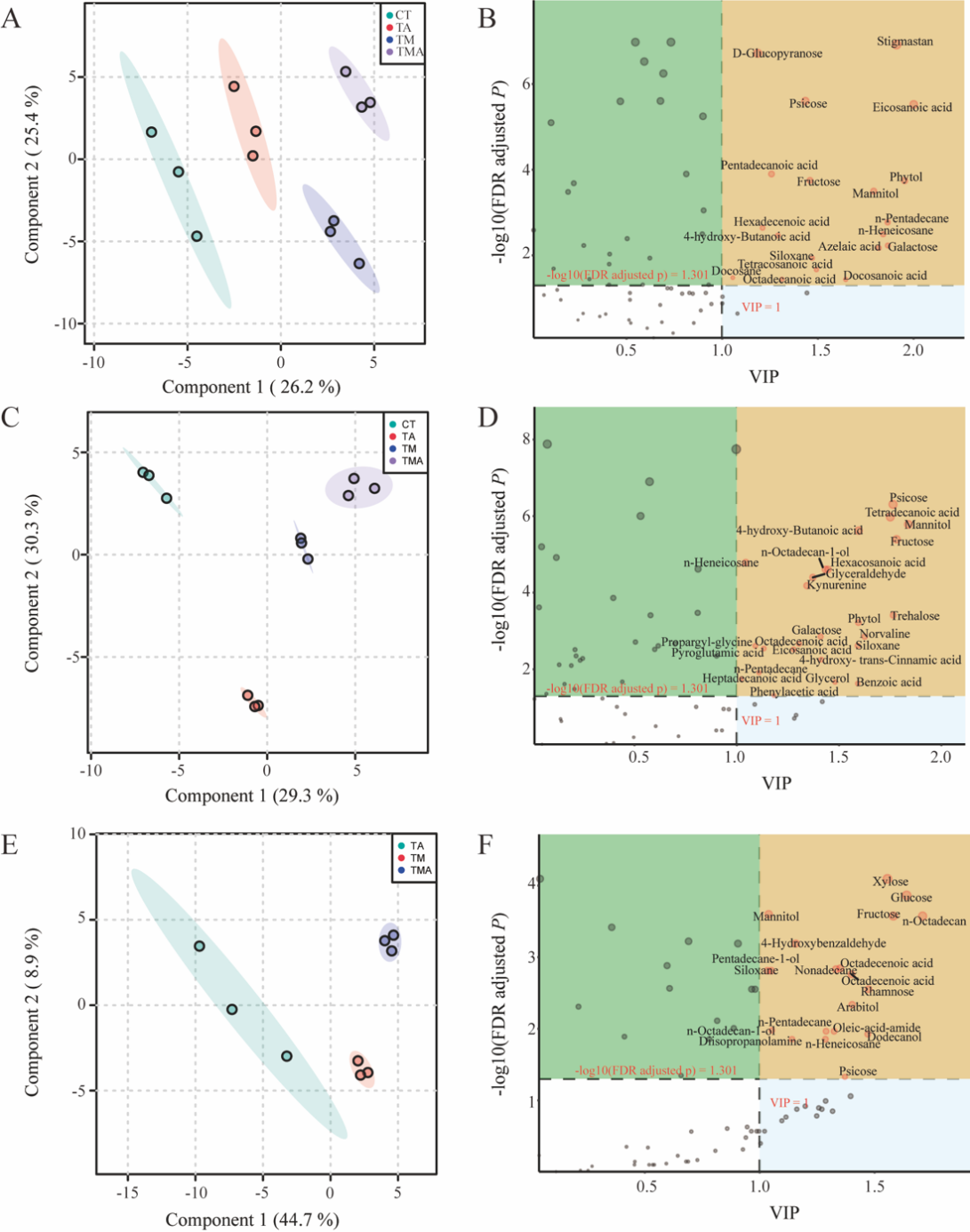


**Supplementary Figure 3.** OPLS-DA of root exudates in intercropping groups. (A) Cross-validation model between intercropping treatment in FRS, BS and ARS, R2X=0.792, R2Y=0.986, Q2Y=0.952. (B) The VIP valus of differential root exudates in FRS. (C) Cross-validation model between intercropping treatment in BS, R2X=0.836, R2Y=0.985, Q2Y=0.949. (D) The VIP valus of differential root exudates in BS. (E) Cross-validation model between intercropping treatment in ARS, R2X=0.841, R2Y=0.994, Q2Y=0.970. (F) The VIP valus of differential root exudates in ARS. R2X and R2Y represent the explanatory power of the constructed model to the X and Y matrices, respectively. Q2 indicates the predictive ability of the model, and the closer their values are to 1, the better the fit of the model.


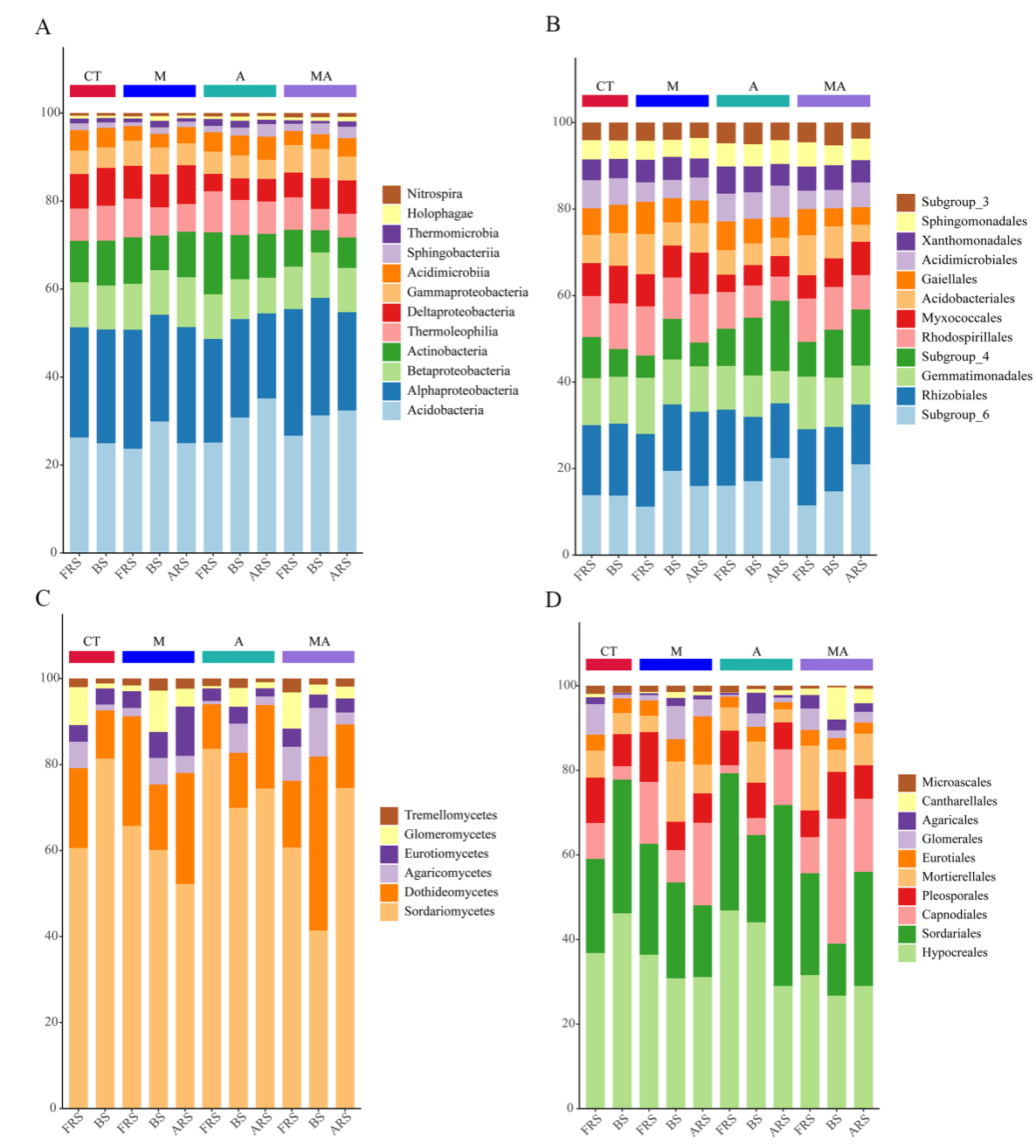


**Supplementary Figure 4.** Percentage of bacterial and fungi community at the taxonomy of class and order. (A) Bacterial community at class level, (B) Bacterial community at order level, (C) Fungi community at class level, (D) Fungi community at order level. (CT, mono-cultured three young annual *M. hupehensis* tree at 10 cm aquilateral triangle; TM, three young annual *M. hupehensis* tree intercropping with ten M. haplocalyx seedlings; TA, three young annual *M. hupehensis* tree intercropping with ten *A. conyzoides* seedlings; TMA, three young annual *M. hupehensis* tree mixed intercropping with five *M. haplocalyx* seedlings and five A. conyzoides. FRS, *M. hupehensis* seedling rhizosphere soil; BS, young annual *M. hupehensis* tree non-rhizosphere soil and aromatic plants seedling non-rhizosphere soil; ARS, aromatic plants seedling rhizosphere soil). The relative abundance of bacterial and fungi community < 1% was filterated.

**Table S1. Differences of root exudates in intercropping groups and soil ecological niche.**

**Table S2. The Sobs, Chao and Shannon indices of the bacteria and fungi.**

|  | Treatment | Root zones | Sobs | Chao | Shannon |
| --- | --- | --- | --- | --- | --- |
| Bacteria | CT | FRS | 6183.00±141.82abc | 9283.06±445.10ab | 7.56±0.06abc |
|  |  | BS | 6359.67±324.15ab | 9499.23±440.17a | 7.62±0.14ab |
|  | TM | FRS | 5957.33±272.95abcde | 9064.91±316.63abc | 7.49±0.13bcd |
|  |  | BS | 5813.6±338.72bcdef | 8518.50±693.23bcd | 7.52±0.09bcd |
|  |  | ARS | 6430.00±178.13a | 9559.08±35.08a | 7.70±0.11a |
|  | TA | FRS | 6018.00±167.49abcd | 8849.23±214.34abc | 7.50±0.08bcd |
|  |  | BS | 5582.67±305.22def | 8466.50±268.18bcd | 7.37±0.09d |
|  |  | ARS | 5382.33±397.15f | 7726.84±805.19d | 7.44±0.07cd |
|  | TMA | FRS | 5747.33±472.73cdef | 8621.28±515.81bc | 7.39±0.12cd |
|  |  | BS | 5448.67±307.31ef | 8347.05±438.93cd | 7.36±0.08d |
|  |  | ARS | 6093.67±32.50abcd | 9148.28±273.64abc | 7.64±0.03ab |
| Fungi | CT | FRS | 776.00±136.07ab | 978.68±252.06ab | 4.46±0.33abc |
|  |  | BS | 859.67±25.03ab | 1137.55±11.30a | 4.17±0.08c |
|  | TM | FRS | 836.33±27.10ab | 1093.17±38.77ab | 4.24±0.17bc |
|  |  | BS | 750.00±167.23ab | 997.42±330.38ab | 4.24±0.36bc |
|  |  | ARS | 868.33±66.71ab | 1201.62±106.97a | 4.06±0.43c |
|  | TA | FRS | 870.67±98.11ab | 1173.89±86.67a | 4.27±0.33abc |
|  |  | BS | 898.33±133.02a | 1125.50±235.58a | 4.82±0.37a |
|  |  | ARS | 733.67±126.08ab | 860.27±233.20ab | 4.56±0.13abc |
|  | TMA | FRS | 829.67±85.58ab | 1087.20±195.35ab | 4.36±0.43abc |
|  |  | BS | 696.00±87.68b | 771.48±115.30b | 4.776±0.18ab |
|  |  | ARS | 765.67±36.47ab | 937.33±92.34ab | 4.38±0.10abc |

Notes: Sobs, number of OTUs observed; Chao, number of OTUs estimated; Shannon, community diversity. TM, young apples intercropping with medium growth-potential *Mentha haplocalyx* Briq.; TA, young apples intercropping with high growth-potential *Ageratum conyzoides* L.; TMA, as well as mixed intercropping with medium growth-potential *Mentha haplocalyx* Briq. and high growth-potential *Ageratum conyzoides* L.; CT, only planted apple trees. FRS, apples rhizosphere soil; BS, relative to soil bulk; ARS, aromatic plant rhizosphere soil. Different letters indicate signifificant differences (P < 0.05) based on Duncan's multiple range test. Values are the mean ±SD (n=3). Different letters indicate signifificant differences (*P* < 0.05) based on Duncan's multiple range test.
